# Cutaneous Alternariosis Caused by *Alternaria infectoria*: Three Cases in Kidney Transplant Patients

**DOI:** 10.3390/healthcare1010100

**Published:** 2013-10-30

**Authors:** Leonor Lopes, João Borges-Costa, Luís Soares-Almeida, Paulo Filipe, Fernanda Neves, Alice Santana, José Guerra, Heinz Kutzner

**Affiliations:** 1 Dermatology Center, Santa Maria Hospital, Avenida Prof. Egas Moniz, Lisboa 1649-028, Portugal; E-Mails: leonorlopes@gmail.com (L.L.); soaresdealmeida@chln.min-saude.pt (L.S.-A.); paulolealfilipe@gmail.com (P.F.); 2 Dermatology Research Unit, Institute of Molecular Medicine, Avenida Prof. Egas Moniz, Lisboa 1649-028, Portugal; 3 Dermatology Center, Oeste Hospital Center, Rua Diário de Notícias, Caldas da Rainha 2500-176, Portugal; E-Mail: fcneves@sapo.pt; 4 Nefrology Center, Santa Maria Hospital, Avenida Prof. Egas Moniz, Lisboa 1649-028, Portugal; E-Mails: alicesantana1@sapo.pt (A.S.); joseoliveiraguerra@gmail.com (J.G.); 5 Dermatopathology Center, Siemensstr. 6/1, Friedrichshafen D88048, Germany; E-Mail: kutzner@w-4.de

**Keywords:** cutaneous alternariosis, *Alternaria infectoria*, infection skin disorders in organ transplant recipients

## Abstract

The genus *Alternaria* has more than 80 species. *Alternaria alternata* and *Alternaria infectoria* are the most frequent species associated with infections in humans. Their clinical importance lies in the growing number of cases reported in immunocompromised patients. Herein, we report three cases of kidney-transplanted patients with different clinical presentations of cutaneous alternariosis and we discuss the treatment options.

## 1. Introduction

*Alternaria* species are dematiaceous fungi which are characterized by the formation of grey to black colonies on culture, due to the production of a melanin-like pigment in the cell wall of hyphae and conidia [1]. Since they are ubiquitous in the environment, their isolation in culture requires careful clinical evaluation [2]. The genus *Alternaria* comprises more than 80 species [3]. *Alternaria alternata* and *Alternaria infectoria* are the most common species in human infections [4,5]. Rarely, they can cause rhinitis, asthma, pneumonia, sinusitis, osteomyelitis, peritonitis, keratitis and granulomatous lung disease [1,3,6]. *Alternaria* cutaneous infection is an infrequent cause of skin infection in immunocompromised patients [1,7]. 

## 2. Results and Discussion

**Figure 1:**
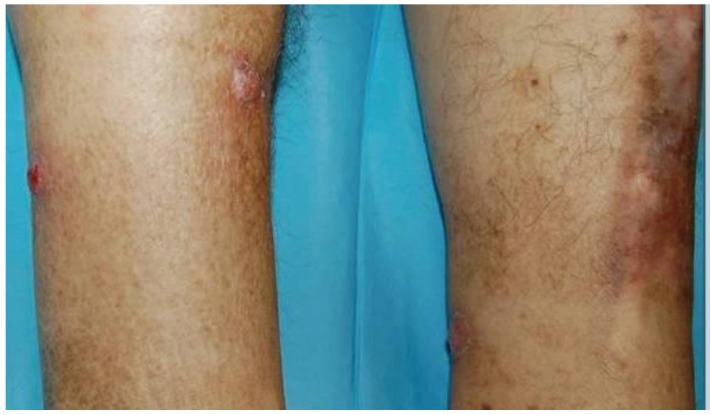
Clinical presentation of cutaneous alternariosis: violaceous nodules on the legs.

Case 1—A 61-year-old Caucasian male gardener was presented with a 2-weeks history of five tender violaceous nodules, with less than two cm in diameter, on both legs (Figure 1). He had been submitted to a renal transplantation six months earlier due to end-stage renal disease associated with diabetes mellitus, and he was medicated with tacrolimus 2 mg twice a day and prednisolone 5 mg once a day. The skin biopsy showed pseudoepitheliomatous hyperplasia of the epidermis and suppurative granulomas in the dermis. Direct examination of biopsy specimen (performed with 10% of potassium hydroxide solution) revealed hyaline septate hyphae. Cultures on Sabouraud Dextrose Agar, incubated at 24–37 °C, developed grayish cottony colonies in all inocula. Microscopic examination revealed dark septate hyphae, non-branched conidiophores and pigmented ovoid conidia. *Alternaria infectoria* was also confirmed by polymerase chain reaction (PCR) from skin biopsy. This method was performed by DNA sequences of nuclear ribosomal internal transcribed spacer (ITS) 1 region. The patient was successfully treated with itraconazole 100 mg daily *per os*, for 3 months without relapses in a 36 months follow-up period.

**Figure 2:**
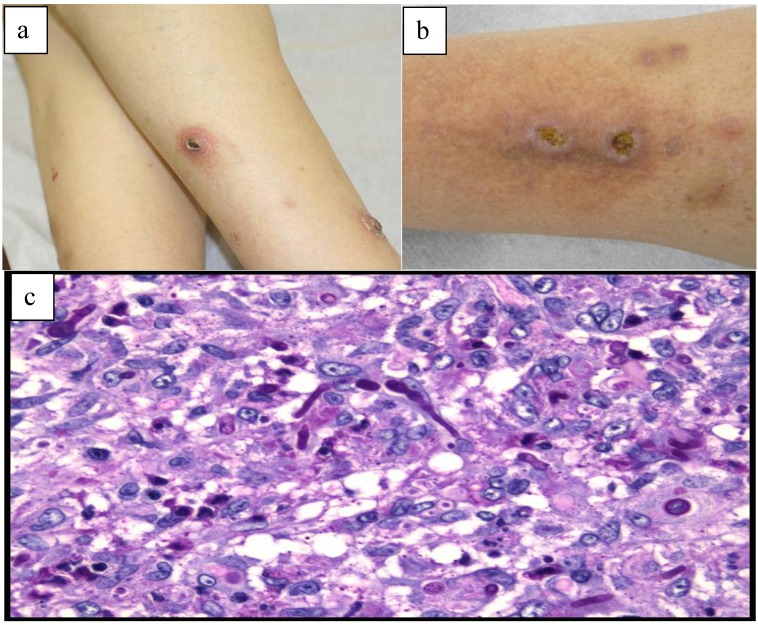
(**a**) Clinical presentation of cutaneous alternariosis: papules and nodules with a fine scaly and a central hemorrhagic crust on both legs; (**b**) Clinical presentation of cutaneousalternariosis: two ulcerated nodules on the right leg; (**c**) Histopathologic findings of cutaneous alternariosis in skin biopsy: presence of septate hyphae on Periodic-acid-Schiff stain.

Case 2—A 63-year-old Caucasian male patient, living in a rural area, with arterial hypertension was referred to us, 14 months after renal transplantation, with multiple, painless, well-circumscribed erythematous papules and nodules with a fine scaly and a central hemorrhagic crust on both legs evolving during one year (Figure 2a). Two nodules were ulcerated on the anterior aspect of the right leg (Figure 2b). The patient denied any local skin trauma since transplantation and he was medicated with prednisolone 10 mg once a day and cyclosporine 100 mg twice daily. Three months earlier, he was submitted to 2 cycles of cryotherapy on the lesions, without improvement. The histopathologic examination revealed the presence of septate hyphae on Periodic-acid-Schiff stain (Figure 2c). Concomitant Sabouraud Dextrose agar cultures showed typical morphology of the fungi, which enabled it to be identified as *Alternaria infectoria.* This result was also confirmed by PCR from skin biopsy. The patient improved gradually after one month of posaconazole 200 mg *per os* daily, but residual hyperpigmentation persisted. At the 12-month follow-up visit, there were no relapses.

**Figure 3:**
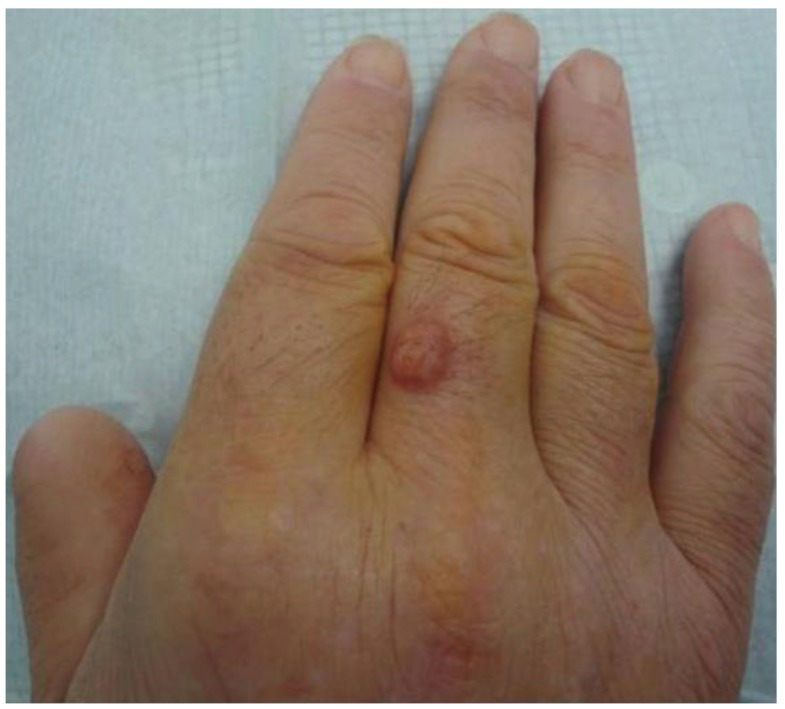
Clinical presentation of cutaneous alternariosis: erythematosus nodule on the dorsal aspect of the third finger of the right hand.

Case 3—A 56-year-old Caucasian man, with a kidney transplant eleven years earlier due to type 2 diabetes mellitus and medicated with prednisolone 5 mg once daily, tacrolimus 2 mg twice a day and mycophenolate mofetil 500 mg twice daily, was admitted with a 5-month history of a hard, painless, erythematosus nodule, with 2 cm in diameter, located on the dorsal aspect of the third finger of the right hand (Figure 3). Due to the rapid growth, surgical excision was performed assuming a tumoral lesion such as squamous cell carcinoma or amelanotic melanoma. The histopathologic examination showed septate hyphae in a granulomatous dermal infiltrate. The *Alternaria infectoria* diagnosis was made only by PCR. The treatment was complemented with itraconazole 100 mg *per os* daily for 3 months. The patient is free of lesions 17-months after surgical excision.

The clinical and mycological findings, treatment and follow-up of the three cases reported are summarized in Table 1.

**Table 1:** Clinical and mycological findings, treatment and follow-up of the three cases reported.

	Sex/Age	Immunosuppression	Medication	Clinical findings	Evolution	Diagnosis	Intervention	Follow-up
**1**	M/61 years	Kidney transplant (6-months prior)	Tacrolimus Prednisolone	Five tender, violaceous nodules on both legs	2 weeks	Histological examination Culture PCR	Itraconazole 100 mg/day, 3 months	36-months No relapse
**2**	M/63 years	Kidney transplant (14-months prior)	Cyclosporine Prednisolone	Papulo-nodules and 2 ulcerations on the legs	1 year	Histological examination Culture PCR	Cryotherapy Posaconazole 200 mg/day, 1 month	12-months No relapse
**3**	M/56 years	Kidney transplant (11-years prior)	Tacrolimus Prednisolone Mycophenolate mofetil	1 nodule on the third finger of the right hand	5 months	Histological examination PCR	Surgical excision Itraconazole 100 mg/day, 3 months	17-months No relapse

*Alternaria* infection has a broad clinical spectrum of cutaneous disease which includes: papules and nodules which can evolve into non-healing ulcers, erythematosus infiltrating patches, verruciform lesions and chronically vegetating tumorous infiltrates [1,8]. The main predisposing factors for this infection are jobs with frequent contact with soil, diabetes mellitus and local skin trauma associated to immunosuppression [1]. 

The diagnosis is based on histopathological findings, mycologic examination and, more recently, with molecular biology methods, such as polymerase chain reaction. Since *Alternaria* can be isolated on normal human skin or as a laboratory contaminant, histological confirmation is essential to establish a good clinical correlation [3,6,8]. This infection can occur at any time after transplantation. However, 70% of the cases occur within the first year [1].

There are only few cases of cutaneous alternariosis due to *Alternaria infectoria*, which may be due to misidentification of this species caused by the frequent lack of sporulation in microscopic examination [5,9]. In our patients, the identification of *Alternaria* spp. was obtained by culture and the identification of the species by PCR. The importance of molecular methods in *Alternaria* cutaneous infection has already been reported [5,9]. We emphasize that this approach may allow an accurate and fast diagnosis for these infections. 

There are no guidelines for the treatment of cutaneous alternariosis. The disease, without treatment, has a fluctuating and chronic course [1]. If possible, reduction of immunosuppression can be effective in the treatment [3,8]. In patients with few and small lesions, surgical excision may be performed [3,10]. When it is not possible to excise the lesions, systemic antifungal treatment may be introduced. Currently, itraconazole is considered the drug of choice due to its low toxicity [3,6,7,8,10]. However, it has interaction with calcineurin inhibitors, which are frequently used in kidney-transplanted patients [7]. The doses of itraconazole range from 100 to 600 mg daily, with an average duration therapy of 2.8 months [6,7,8]. Other options such as: fluconazole, terbinafine, amphotericin B, voriconazole and posaconazole have also been used [7,8,11]. Some authors consider itraconazole as effective as amphotericin B in the treatment of these infections [12]. In recent studies, posaconazole was considered superior than itraconazole because it has lower minimal inhibitory concentrations, better distribution and less drug interactions [11]. However, there are limited data for its use [6]. Cryotherapy may be an option in the treatment of multiple lesions, when surgical excision is not possible [7,8]. The best therapeutic option for these patients is the combination of surgical excision with systemic antifungal treatment [1,12]. The reduction of immunosuppressant drugs in the maintenance phase of transplanted patients is not always possible due to the risk of graft rejection. We used antifungal systemic treatment with itraconazole alone; or combined treatments with cryotherapy and posaconazole or surgical excision with itraconazole. Despite clinical improvement, relapses are frequent. In our series, relapses with either of these therapeutic options were not observed. However, relapses can occur even after prolonged treatment [3]. Therefore, long-term clinical follow-up is advised [3,8,13]. 

## 3. Conclusions

As the number of patients under immunosuppressive drug therapy and their survival time increases, the risk of these cutaneous infections is also likely to increase [10]. Combined treatment options and the development of new systemic antifungal therapies may improve the treatment. Large series are needed in order to decide the best therapeutic option for these patients.
